# Expression of proto-oncogene *KIT* is up-regulated in subset of human meningiomas

**DOI:** 10.1186/1471-2407-12-212

**Published:** 2012-06-06

**Authors:** Masum Saini, Ajaya Nand Jha, Andleeb Abrari, Sher Ali

**Affiliations:** 1 Molecular Genetics Laboratory, National Institute of Immunology, Aruna Asaf Ali Marg, New Delhi, 110067, India; 2 Max Super Specialty Hospital, 1, Press Enclave Road, Saket, New Delhi, 110017, India

## Abstract

**Background:**

*KIT* is a proto-oncogene involved in diverse neoplastic processes. Aberrant kinase activity of the KIT receptor has been targeted by tyrosine kinase inhibitor (TKI) therapy in different neoplasias. In all the earlier studies, KIT expression was reported to be absent in meningiomas. However, we observed *KIT* mRNA expression in some meningioma cases. This prompted us to undertake its detailed analyses in meningioma tissues resected during 2008–2009.

**Methods:**

Tumor tissues and matched peripheral blood samples collected from meningioma patients were used for detailed molecular analyses. KIT expression was ascertained immunohistochemically and validated by immunoblotting. *KIT* and *KITLG* transcript levels were discerned by reverse transcription quantitative real-time PCR (RT-qPCR). Similarly, *KIT* amplification and allele loss were assessed by quantitative real-time (qPCR) and validated by fluorescence *in situ* hybridization (FISH) on the neoplastic tissues. Possible alterations of the gene at the nucleotide level were analyzed by sequencing.

**Results:**

Contrary to earlier reports, KIT expression, was detected immunohistochemically in 20.6% meningioma cases (n = 34). Receptor (*KIT)* and ligand (*KITLG)* transcripts monitored by RT-qPCR were found to co-express (p = 0.048) in most of the KIT immunopositive tumors. 1/7 KIT positive meningiomas showed allele loss corroborated by reduced FISH signal in the corresponding neoplastic tissue. Sequence analysis of *KIT* showed M541L substitution in exon 10, in one of the immunopositive cases. However, its biological consequence remains to be uncovered.

**Conclusions:**

This study clearly demonstrates KIT over-expression in the human meningiomas. The data suggest that up-regulated *KIT* transcription (p < 0.001), instead of gene amplification (p > 0.05), is a likely mechanism responsible for altered KIT expression. Thus, *KIT* is a potential candidate for detailed investigation in the context of meningioma pathogenesis.

## Background

Genetic alterations causing deregulated expression of oncogenes and tumor suppressor genes underlie most of the neoplastic events. Receptor tyrosine kinases (RTKs) constitute a discrete category of oncogenes and are integral molecules of signaling cascades. Their aberrations and deranged cross-talks lead to pathological conditions [1]. *KIT* (CD117*, SCFR*) a proto-oncogene on human chromosome 4q12, encodes one such transmembrane RTK of type III receptor family [2,3]. Its pleiotropic attribute is well established as the gene is involved in normal growth and developmental processes [4-7]. *KITLG* (*KIT* Ligand/*SCF*-12q22) is the ligand of KIT receptor [8]. In the normal human adult, a striking regional expression pattern for KIT and KITLG is seen in the central nervous system (CNS). Normal meninges are KIT and KITLG immunonegative with interspersed KIT positive mast cells [9]. KIT stimulation by KITLG also triggers oncogenic signaling pathways resulting in unrestricted proliferation, cell survival, migration and apoptosis [10]. Hypoxia in the tumor microenvironment induces KITLG secretion furthering neovascularisation and promoting tumor sustenance [11].

Aberrant KIT expression is reported in gastrointestinal stromal tumors (GISTs), acute myeloid leukemia (AML), small cell lung carcinoma, breast cancer, gliomas and neuroendocrine tumors [12]. GISTs with KIT over-expression showed mRNA levels ranging from 0.01 to 5.8 folds, based on RT-qPCR [13]. In GISTs, oncogenic activation of KIT is the frequent pathogenic mechanism and hence its expression serves as a diagnostic biomarker. KIT positive cases, bearing its activating mutations show a spectacular response to inhibition therapy with imatinib [14]. Imatinib mesylate (STI-571) or Gleevec® is a selective TKI developed to target ABL (Abelson kinase) in BCR-ABL fusion oncoprotein in CML patients. It was fortuitously found to inhibit other kinases such as PDGFR (alpha- and beta-platelet-derived growth factor receptors) and KIT, in GISTs and other malignancies [15]. In view of the above, evaluation of the *KIT* for its role in tumors of the CNS seems to be a clinically rewarding proposition.

Meningiomas are mesenchymal tumors originating from the meninges. Based on the degrees of malignancy, these tumors are graded as benign (WHO grade I), atypical (WHO grade II) and anaplastic/malignant (WHO grade III) [16]. Overall, meningiomas are neoplasms where the benign forms exert their devastating effects through volume expansion in confined regions of the brain. Besides producing increased intracranial pressure, the malignant forms are associated with brain invasion, early recurrence and decreased survival rates. At times, their location in the brain is critical, such that they press upon important faculties and show tenacity even to surgical intervention [17]. In view of this, alternate therapeutic approaches are being explored to address these challenges.

Meningiomas have been reported to lack KIT expression in three independent studies [18-20]. Of these, one on KIT expression in germinomas randomly included a single meningioma sample [19]. In the second one on human solid tumors, 8 meningioma cases were included [18]. The third study focused on the analysis of KIT immunoexpression in 37 meningiomas and reported lack of its expression [20]. Clinical trials were undertaken with imatinib singly or in combination with hydroxyurea, in recurrent meningiomas [21,22]. These trials were based on the reports that implicated co-expression of PDGF and PDGFR in autocrine growth stimulation of meningioma cells. One of the trials was closed prematurely due to slow accrual. Further, due to insufficient number of samples available for validating PDGFR expression, its correlation with imatinib treatment could not be established [21]. The second trial reported the combination therapy to have modest anti-tumor activity [22]. The biopsies of patients enrolled in these trials were not profiled for possible KIT expression/alterations.

Despite reported absence of KIT expression in meningiomas, our initial observation of its mRNA expression (by RT-PCR) in some cases (Additional file 1A) evoked our interest to ascertain its status in the present study. We indeed observed up-regulated *KIT* protein and mRNA expression in a subset of meningiomas.

## Methods

### Sample collection

The protocols followed in the present study were approved by both, the National Institute of Immunology’s Institutional Human Ethics Committee and the Max Healthcare Ethics Committee. A series of 34 patients operated consecutively for primary intracranial meningiomas during May 2008-August 2009 at the Max hospital’s Neurosciences department was included in this study. Parts of the resected tumor tissues and matched peripheral blood samples were collected from meningioma patients with their written informed consents. The samples were taken at the first diagnosis of meningioma without further selection. Histopathological examination and grading of tumors were performed following WHO guidelines 2007 [16]. Of the 34 meningioma (M) samples collected, 24 were from females and 10, males. Case M29 was diagnosed with multiple meningiomas (right frontal and cervical), but the intracranial right frontal one was surgically resected and analyzed in this study. Tables 1 and 2 summarize the grade wise distribution of the cases analyzed in the present study and details of KIT immunopositive meningioma cases, respectively.

**Table 1:** Grade wise distribution of meningioma cases analyzed

**Meningiomas**	**Grade I (Varaint/Sub-type)**					**Grade II**	**Grade III**	**Total**
	**Ag**	**X**	**Mg**	**Fb**	**T**	**At**	**Ap**	
**Cases analyzed**	1	1	15	6	7	3	1	34
**KIT positive cases**	–	–	2 (13.3%)	1 (16.7%)	3 (42.9%)	1 (33.3%)	–	7 (20.6%)

(Ag, Angiomatous; At, Atypical; Ap, Anaplastic; Fb, Fibroblastic; Mg, Meningothelial; T, Transitional; X, Xanthomatous).

**Table 2:** Clinico-pathological and KIT-IHC details of the immunopositive meningioma cases

	**Details**	**Meningioma Cases**						
		**M10**	**M14**	**M15**	**M16**	**M21**	**M29**	**M37**
**Clinico-pathological details**	**Age**	79	48	59	32	51	44	49
	**Gender**	Ml	Fl	Fl	Fl	Fl	Fl	Fl
	**Grade**	I	I	I	I	I	II	I
	**Variant/sub-type**	Mg	T	Mg	T	Fb	At	T
	**Tumor location**	RSW	LPP	PS	FM	LP	RF	LO
	**Recurrent case**	–	–	–	–	–	–	–
**Details of KIT-IHC analyses**	**% positivity**^**a**^	4	2	2-4	1	4	3	1
	**Staining intensity**^**b**^	W	Md	W-Md	W	S	S	W
	**Stain localization**^**c**^	C	C	C	C	C	C	C
	**Staining pattern**^**c**^	D,G	F,G	H,G	H,G	D	D	F,G

(**Clinical data**: Ml, Male; Fl, Female. Ag, Angiomatous; At, Atypical; Ap, Anaplastic; Fb, Fibroblastic; Mg, Meningothelial; T, Transitional; FM, Foramen magnum; LO, Left occipital; LP, Left Parietal; LPP, Left parietal parasaggital; PS, Planum sphenoidale; RF, Right frontal; RSW, Right sphenoid wing.

**Experimental data:**^**a**^ 0 = 0%, 1 + = 1–10%, 2 + = 11–50%, 3 + = 51–75%, 4 + = ≥ 75.

^**b**^ W, Weak (+); Md, Moderate (++); S, Strong (+++).

^**c**^ C, Cytoplasmic; D, Diffuse; F, Focal; G, Granular; H, Homogeneous.

### Isolation of genomic DNA, total RNA and cDNA synthesis

DNA extraction kit (Qiagen, Valencia, CA, USA) was used to isolate DNA from peripheral blood leukocytes (PBLs) as per the manufacturer’s protocol. Tumor tissue portions collected in RNA*later* (Ambion, Austin, TX, USA), were used for DNA isolation by phenol chloroform extraction method, following standard protocol [23]. Total RNA isolation from tumor tissues was performed using Tri Reagent RT (Molecular Research Centre, Cincinnati, OH, USA), as recommended by the manufacturer. Quality of isolated DNA and RNA was evaluated using 1% agarose and 1% denaturing agarose gels, respectively. Concentration and purity of the nucleic acids were confirmed using the spectrophotometer. Potential DNA contamination of total RNA was checked using *GAPDH* primers in a 20 μl reaction volume of PCR. Subsequently, RNA was reverse transcribed using High Capacity cDNA Archive kit (ABI, Carlsbad, CA, USA). PCR was conducted using *ACTB* primers for assessing the quality of synthesized cDNA (Additional file 1B). Primers for the cytoplasmic domain of *KIT* were used to detect the gene’s transcripts in the samples by RT-PCR (Additional file 1A). Primer details have been given in Additional file 2.

### Immunohistochemical staining

Serial sections of formalin fixed paraffin embedded (FFPE) tumor tissues used for histopathological diagnosis were evaluated for KIT expression using immunoperoxidase-diaminobenzidine (HRP-DAB) staining. Differences in KIT expression in other types of cancers have been attributed to properties of the antibody and staining procedure used for IHC. Therefore, in the present study anti-human KIT antibody from DAKO (A4502), reported to have higher sensitivity was used [24,25]. Briefly, 4 μm thin sections were de-waxed, washed through graded ethanol series and rehydrated under running water. The staining was performed by careful optimization of the vendor’s recommendations. Heat induced epitope retrieval (HIER, previously optimized) was performed in boiling 1:0.1 mM Tris-EDTA buffer pH 9.0 in a pressure cooker for ~3 minutes (Pattern of KIT staining without HIER was also found to be similar in our hands). Tumoral tissue sections (TTS) were treated with peroxidase block (3% H_2_O_2_) for 15 minutes. Samples were then sequentially incubated with polyclonal rabbit anti-human KIT antibody (A4502, DAKO, Carpinteria, CA, USA, optimized dilution 1:100) for 60 minutes, DAKO EnVision + System-HRP labelled polymer for 30 minutes and DAKO liquid DAB + substrate chromogen system for 10 minutes, in a humid chamber at room temperature. Following each incubation step, TTS were rinsed in 1xTBS (pH 7.6) thrice for 2 minutes. They were counter stained in Harris haematoxylin for 2 minutes, dehydrated in an ethanol series, air-dried and mounted in DPX. To ensure specificity of the antibody, appropriate controls, such as uterine tissue (negative control), GIST (characterized KIT positive case) from the clinical collaborators and commercially purchased normal meningeal tissue sections (BioChain, Hayward, CA, USA) were included for processing in each batch. Additionally, a negative control omitting the primary antibody was included. The TTS were evaluated for staining using light microscopy, by two pathologists to account for inter-observer variability. The pathologists were unaware of the genetic analyses. Immuno-stained tissues were imaged on 1X51 microscope (Olympus, Tokyo, Japan) equipped with U-CMAD 3 camera (Olympus) operated by DP Controller software v3.1.1267 (Olympus) and Image Pro Express 6.3 software (Media Cybernetics, MD, USA). Presence of KIT immunostain was evaluated for criteria such as, percentage positivity (0 = 0%, 1 + = 5–10%, 2 + = 11–50%, 3 + = 51–75%, 4 + = ≥ 75%), location (cytoplasmic, nuclear, fibrillar), pattern (diffuse, focal, granular, homogeneous) and intensity (weak; +, moderate; ++, strong; +++). To ensure reproducibility of results, IHC was repeated twice for the KIT positive cases.

### Immunoblotting

Cases, where adequate tumor tissues were available, the IHC findings were confirmed by western blotting. For this, tumor tissues were lysed in RIPA lysis buffer and separated by centrifugation. Commercially purchased total proteins of relevant non-neoplastic tissues were used as controls (Biochain). After resolving the proteins on 12% SDS polyacrylamide gels, they were transferred onto nitrocellulose membranes (Millipore, Billercia, MA, USA). The membranes were blocked in PBS buffer supplemented with 3% non-fat dried milk and 2% BSA (Cell Signaling Technology, Boston, MA, USA). Membranes were probed using polyclonal rabbit anti-human KIT (A4502, DAKO, dilution 1:1500) and polyclonal rabbit anti-Beta Tubulin (RB-9249-P1; Neo Markers, Thermo Fisher Scientific, Fremont, CA, USA, dilution 1:10,000) as primary antibodies. The proteins were detected using horse-radish peroxidase conjugated goat anti-rabbit antibody (111-036-045, Jackson Immuno Research Laboratories Inc., West Grove, PA, USA, dilution 1:10,000) and immobilon western chemiluminescent HRP substrate (Millipore).

### Real-time quantification of *KIT* and *KITLG* transcripts

Primers for the genes of interest (GI) and reference (GR) were designed using Primer Express 3.0 software (ABI). Specificity of the primers was confirmed by blastn algorithm of the BLAST program. *GAPDH* was used as an endogenous control or GR, as it showed stable expression in both control and test samples. SYBR® green (ABI) assays were performed for relative quantification of *KIT* and *KITLG* transcripts on 7500 Real-Time PCR System (ABI). Commercially purchased total RNA of human ♂ meninges and ♀ dura were used as controls or calibrators (BioChain and Clontech, Mountain View, CA, USA; respectively). Two fold dilution series of the cDNA template were assayed to generate standard and disassociation curves. Standard curves of primers, for all the three genes, had slope values within the range of −3.3 to −3.4, R^2^ >0.99 (co-efficient of determination) confirming comparable PCR efficiencies and good fit of data points, respectively. Further single melting curve peaks validated specific amplification (data not shown). Standard curve was employed to ascertain the amount of sample cDNA to be used for RT-qPCR. All the assays were performed using respective gene’s 100 nM of forward and reverse primers, in a final reaction volume of 20 μl. Universal cycling conditions as recommended by ABI were employed to amplify GIs and GR in separate wells. The results were ratified, when of the triplicate Ct values (Cycle threshold), at least two were concordant. Expression levels were calculated by the relative quantification (RQ = 2^−ΔΔCt^) method [26]. ΔΔCt is the cycle threshold normalized first with the endogenous control (ΔCt = Ct GI - Ct GR) and then with the calibrator sample (ΔΔCt = ΔCt Sample - ΔCt Calibrator). SDS 7500™ Software v2.0.3 was used to analyze the data and heat map was generated using Data Assist™ Software v2.0 (ABI).

### Copy number assessment of *KIT* by qPCR

Copy number of *KIT* was assessed using a commercial pre-designed TaqMan® assay (Hs02812715_cn; ABI) and *RNase P* as the reference gene (TaqMan® *RNase P* detection kit P/N: 4316831, ABI) on 7500 Real-Time PCR System (ABI). Tumor DNA comprised the test samples. DNA from matched PBLs of respective patients was also assayed, as endogenous genetic complement to ascertain whether copy number variations are *de novo* neoplastic events. Commercially purchased human brain DNA (BioChain), two genomic DNA samples (provided with the *RNase P* detection kit, ABI) and blood DNA from 2 healthy volunteers served as controls. Universal cycling conditions recommended by ABI were used to amplify all samples and controls in triplicates. The reactions were set up as 20 μl singleplex assays, using 20 ng of genomic DNA, 1x TaqMan® Universal PCR Master Mix (ABI) and 1x primer probe mix (ABI). A normal sample theoretically has two copies of *KIT*/diploid genome. Therefore, gene copy number per diploid genome was calculated using the equation 2 x (2^−ΔΔCt^) [26]. During the study, all the possible measures were taken to avoid non-neoplastic DNA contribution to the test samples. To substantiate the qPCR findings and to rule out experimental errors resulting from potential non-neoplastic cell contribution, FISH was performed on FFPE tissue sections of KIT immunopositive cases.

### Fluorescence *in situ* hybridization

Dual-colour FISH was performed to evaluate *KIT* status and Chromosome 4 ploidy. For this purpose, 4 μm thin serial sections were used from the FFPE tissue blocks, employed earlier for diagnosis and KIT IHC. Moreover, FISH was conducted on primary tumor specimens, instead of cultured tumor cells, to rule out any genetic alterations brought about by *in vitro* manipulation. Commercially purchased normal brain cerebellar tissue section (BioChain) was used as control. Pre-treatment of TTS was performed as described earlier [27]. Subsequently, co-hybridization was conducted using bacterial artificial chromosome (BAC) clone; RP11-586A2 (*KIT*- 4q12; BACPAC Resource CHORI, Oakland, CA, USA) and spectrum green-labelled Chromosome 4 enumeration probe (CEP®4, Vysis, Il, USA) as per manufacturer’s specifications. The BAC clone was nick translated with Texas Red-12-dUTP (Invitrogen, Carlsbad, CA, USA). After hybridization, the nuclei were counterstained with DAPI (Vector Labs, Burlingame, CA, USA).

The slides were viewed on a BX51microscope (Olympus) and approximately 200 non-overlapping tumor-cell nuclei were evaluated from each case. U-TV1X-2 camera fitted on the microscope and CytoVision^TM^ and Genus^TM^ imaging softwares (Applied Imaging, CA, USA) were used to capture the images. A *KIT*/nucleus signal ratio of ≥ 2.5 or *KIT/*CEP4 signal ratio of ≥ 1.5 was considered as *KIT* copy number gain. To confirm the identity of the BAC (RP11-686A2), used for FISH, the clone was streaked to obtain single colonies on LB agar with recommended antibiotics. Of these single colonies, 5 were randomly screened by PCR (Additional file 3) and sequenced (data not shown) using primers specific for portions of each domain of *KIT*.

### Genomic sequence analysis of *KIT* exons

Based on relevant literature review, *KIT* exons were selected for mutational screening. PCR was performed with 50 ng of tumor and matched blood DNA as template from the KIT positive cases in a reaction volume of 35 μl, using primers specific for exons 1, 9, 10, 11, 12, 13 and 17 of *KIT*. Commercially purchased human brain (BioChain) and blood DNA from a healthy volunteer were used as controls. The amplicons were electrophoresed on a 1% agarose gel and purified using QIAquick gel extraction kit as per manufacturer’s guidelines (Qiagen). Sequencing (2X) was conducted with these purified PCR fragments as templates using Big Dye® Terminator v3.1 chemistry (ABI) and standard protocol, on 3130*xl* Genetic Analyser (ABI). Sequences were screened for mutations and compared with corresponding human reference *KIT* exon sequence at NCBI, using Sequence Analysis software v5.3.1 and SeqScape® software v2.6 (ABI).

### Statistical analysis

Sigma Plot 11.0 (Systat Software Inc., Germany) was used to analyze frequency tables employing *χ*^2^ test or Fisher’s exact test. Microsoft Excel 2007 was used to generate scatter plots. The Pearson product–moment correlation coefficient (r), p values and significance of correlation of the plots were determined using Sigma Plot 11.0.

## Results

### KIT immunoexpression is detected in the neoplastic meninges

**Figure 1:**
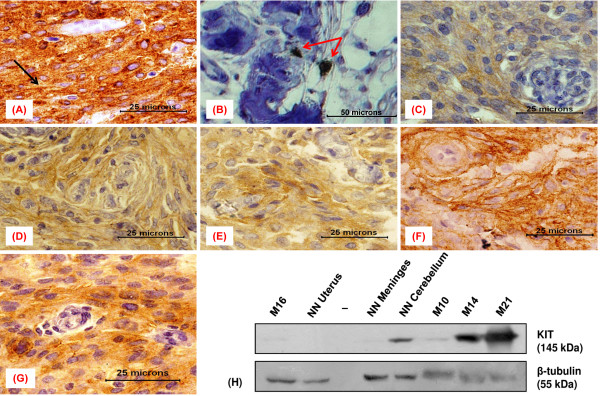
**Immunoexpression of KIT in meningioma and control samples. (A-G)** Immunohistochemical staining results **(A)** GIST (positive control) showing strong immunopositivity with membranous staining (black arrow), **(B)** an otherwise KIT negative meningioma showing interspersed positive mast cells (red arrows) representing an internal control, **(C)** meningothelial meningioma (M10) showing weak granular cytoplasmic staining, **(D)** transitional meningioma (M14) displaying moderate focal staining, **(E)** a meningothelial meningioma (M15) with weak to moderate cytoplasmic KIT staining, **(F)** fibroblastic meningioma (M21) showing strong staining of the cytoplasm, **(G)** an atypical meningioma (M29) with strong cytoplasmic KIT expression. **(H)** Immunoblots of neoplastic and non-neoplastic (NN) tissue lysates, probed with antibodies to KIT and β-Tubulin.

KIT immunopositivity was detected in 20.6% (7/34) meningioma cases (Table 1, Figure 1C-G) as opposed to the earlier report of its negligible expression in 37 meningioma cases [20]. The details of KIT staining intensity, pattern, localization and percentages of immunopositive cells are given in Table 2. Moderate (++) KIT staining was seen in 2/7 meningioma cases, while, strong (+++) expression was detected in 2/7 cases.

Specificity and sensitivity of the anti-KIT antibody and efficacy of the staining protocol were confirmed by the relevant staining patterns observed in the characterized KIT positive and negative control tissues. GIST showed a promisingly strong KIT immunostaining in the cytoplasm with foci of membranous staining (Figure 1A, black arrow). The uterine tissue was indeed KIT negative and presented a clear background (IHC staining not shown). Further, interspersed KIT positive mast cells in an otherwise KIT negative meningioma served as an internal control (Figure 1B). The western blot results were in accordance with that of the IHC observations (Figure 1H). The two immunoassays conducted in the present study used the common antibody to detect KIT, thereby verifying its sensitivity.

### *KIT* and *KITLG* mRNA are co-up regulated in the immunopositive meningiomas

**Figure 2:**
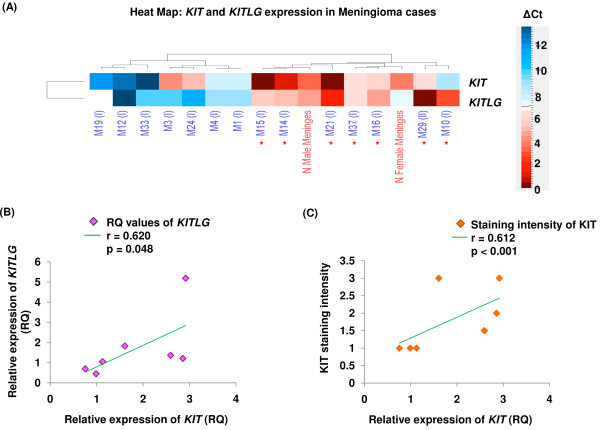
**RT-qPCR estimation of*KIT*and*KITLG*in immunopositive and negative tumor cases**. **(A)** Heat map demonstrating hierarchical clustering of meningioma cases based on ΔCt values. Red asterisk indicates KIT immunopositive cases; representative KIT negative cases are also shown. Scatter plots showing extent of correlation between **(B)***KITLG* and *KIT* mRNA expression, and **(C)** intensity of KIT staining and its relative transcript expression in immunopositive meningioma cases, respectively.

In the present study, heat maps were generated using Euclidean distance and complete linkage to hierarchically cluster meningioma cases based on ΔCt values of *KIT* and *KITLG* expression (Figure 2A, for ΔCt calculation see methods). ΔCt values below and above the mean value indicate up-(red) and down-regulation (blue), respectively (ΔCt scale insert in Figure 2A).

KIT immunopositivity was significantly associated with up-regulated transcript levels in meningioma (p < 0.001 for pair wise comparison, Figure 2C). In the immunopositive meningiomas with up-regulated *KIT* transcripts, RQ values were found to be in the range of 1.13 to 2.92 (for RQ calculations, see methods). Further, *KITLG* transcripts showed significant co-expression with its receptor in immunopositive cases (p = 0.048, Figure 2B). Up-regulated *KITLG* expression showed RQ values ranging from 1.05 to 5.19 in the KIT immunoreactive cases.

### A possible correlation of FISH and qPCR amplification

**Figure 3:**
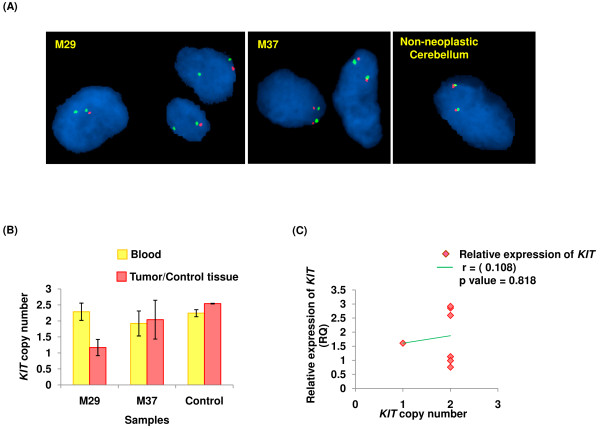
**Copy number status of*KIT*and its correlation with expression. (A)** dual-colour FISH showing *KIT* signal (red) and centromeric sequences on chromosome 4 (green) on tissue sections (100x). M37 and non-neoplastic cerebellum (control) show normal, M29 shows decreased *KIT* copy number. **(B)** qPCR based *KIT* copy number results of meningioma cases; whose FISH micrographs have been shown in (A). **(C)** scatter plot showing relative expression of *KIT* Vs its copy number in immunopositive meningioma cases.

To determine whether an increase in the *KIT* copy number contributes to its over-expression, we undertook its quantification studies. Interestingly, of the 7 KIT positive meningiomas, none showed copy number gain of this gene. Similar observations of KIT over-expression without gain in the gene’s copy number have been reported in GISTs and paediatric renal tumors [13,28]. Notably, M29 tumor tissue showed copy number value of 1.2 (Figure 3B) and 95% of the cells showing *KIT*/nucleus fluorescence signal ratio of ≤ 1.4, suggesting loss of *KIT* allele, akin to hemizygosity s (Additional file 4). This corroborated with a single fluorescing signal for *KIT* (red) in the interphase nuclei (Figure 3A). Observed loss of *KIT* allele in the corresponding case primarily represented neoplastic event since the matched blood sample was found to have 2 copies of *KIT* (Figure 3B, Additional file 4). All the other cases, except the one discussed here showed normal *KIT* copies in the tumor tissues and their constitutional counterparts. *KIT* expression (RQ) plotted against the gene’s copy number showed no significant correlation between the two parameters (p > 0.05, Figure 3C).

### Sequencing of *KIT* shows nucleotide variation in a single immunoreactive case

**Figure 4:**
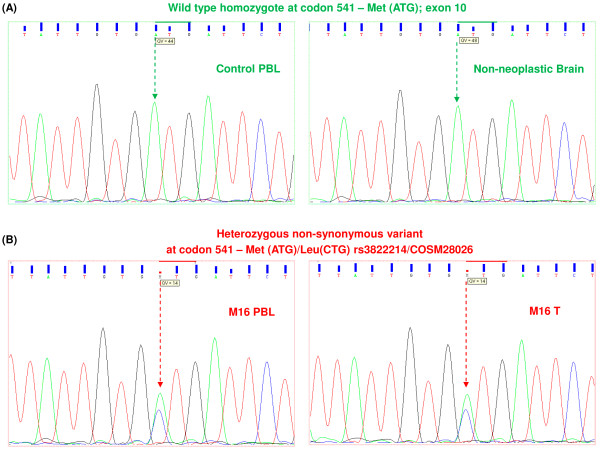
**Electropherograms displaying mutation screening of*KIT*exon 10. (A)** Expected reference allele pattern (green arrows) in DNA from PBL and normal brain “A” (green peak) at first position of codon 541 (exon 10). **(B)** Heterozygous allele pattern (red arrows) showing substitution in DNA from *KIT* positive tumor and matched blood sample in M16. PBL refers to peripheral blood leukocytes, T signifies tumor tissue.

**Figure 5:**
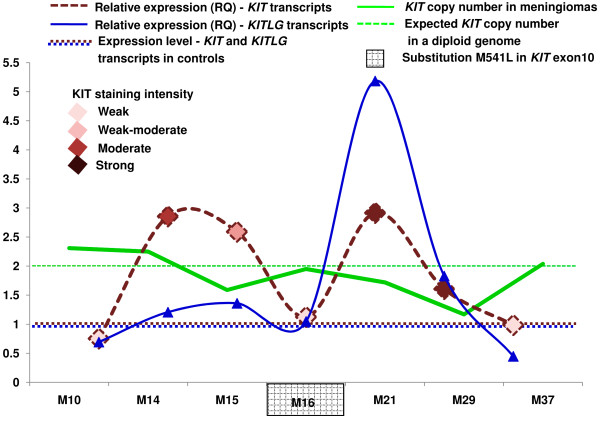
**Summary of*KIT*analyses in the immunopositive meningiomas.**

Analysis of *KIT* in the immunopositive meningioma cases did not reveal loss or gain of function mutations as reported in other pathological conditions. Particularly, though 1/7 meningioma KIT positive cases showed a transversion (A → C) in exon 10, leading to a mis-sense substitution, (Met → Leu) at codon 541(M541L) (Figures 4B and 5, Additional file 4). The variant is an acknowledged reference SNP, rs3822214 in the SNP database dbSNP 135 and is also reported by the Catalogue Of Somatic Mutations in Cancer database (COSMIC) as COSM28026 [29,30]. Figure 4A represents the normal allele pattern as seen in the genomic DNA of non-neoplastic brain and control PBLs.

The variant was observed along with the normal allelic nucleotide in the electropherograms of tumor tissue and matched blood sample of the corresponding patient (Figure 4B, Additional file 4). Therefore, it can be construed that the variation has been stably inherited and is not a *de novo* neoplastic aberration. This SNP having minor allele frequency has been reported by other groups, however its clinical significance is unknown [30].

## Discussion

Contrary to earlier reports of absence of KIT expression in meningiomas, the present study showed its expression in 20.6% cases [18-20]. Of these studies, the one on human solid tumors analyzed only 8 meningioma samples using the DAKO antibody we used in the present study but the staining protocol was not described in sufficient details [18]. The second study was conducted on germinomas using the same antibody and similar protocol for KIT detection as those used in the present study. However, only one meningioma sample was examined [19]. The third study systematically evaluated KIT expression in 37 meningiomas employing a similar staining protocol but a different anti-KIT antibody (MBL, Nagoya, Japan) [20]. Therefore, it is difficult to attribute specific reason(s) to the observed deviation or to compare present study with the earlier ones.

We selected the anti-KIT antibody (DAKO) over other commercial antibodies based on its wide spread usage by different groups working on closely related fields [25,31-36]. Nevertheless, wide variations are practised with regard to antigen retrieval methods and reagents used; and therefore a consensus staining protocol is yet to be established. Employing HIER, the DAKO antibody showed reduced KIT staining in soft tissue sarcomas, however staining of GISTs remained unaffected with or without antigen retrieval [25,35]. Further, there are conflicting reports about effect of antigen retrieval on KIT staining observed in desmoids tumors [25,35,36]. Therefore, in absence of a standard protocol, especially for meningiomas, the vendor’s specifications were followed with careful optimization in the present study. It is noteworthy that the relevant controls showed appropriate staining patterns and the KIT staining results were substantiated by immunoblotting using the same anti-KIT antibody (DAKO).

Graded co-expression of KIT and Zonulin (marker for degraded blood brain barrier) in different grades of brain tumors (5 GBMs and one case each of astrocytoma WHO III and meningioma WHO III) was reported earlier [37]. A meningioma WHO I used as control lacked KIT expression but showed some Zonulin expression. Further, by co-staining Zonulin with a marker for blood vessels (GSI), it was demonstrated that the blood brain barrier was degraded in meningioma WHO III unlike meningioma WHO I. The current finding on the single meningioma grade III being KIT negative reflects a chance occurrence. Use of additional grade III tumor samples for KIT expression analyses would fully resolve this issue.

*KIT* and/or *KITLG* expression quantification in meningiomas was not performed thus far. To the best of our knowledge, data regarding KIT/KITLG expression in the context of meningioma cell lines is not available in public expression database (Array Express on EMBL-EBI). Reportedly, endogenous KITLG co-expression leads to activation of KIT receptors in glioma cell lines and other cancers [38-40]. In the present study, quantification of *KIT* and *KITLG* transcripts revealed significant co-expression in the immunoreactive cases (Figures 2B and 5). Further, with no discernible activating *KIT* mutations in immunopositive meningioma cases, the receptor could possibly get activated via autocrine and/or paracrine modes in these tumors.

Remarkable concordance was observed between KIT immunoexpression and elevated *KIT* transcript levels (Figures 2C and 5). Incidentally, correlation between protein and mRNA expression levels was not always corroborative as in the cases of M10 and 37 (Figure 5). It needs to be determined whether genetic, post-transcriptional or translational mechanisms; regulated in turn by molecules governing KIT expression, underlie these observations. Similar discordance between expression of gene at the transcript and protein levels has been reported earlier [41]. Interestingly, case M29, despite loss of *KIT* allele in the tumor tissue showed strong immunostaining and 1.61 fold higher levels of its transcript (Figure 5, Additional file 4). This could be due to: i) altered pre-transcriptional regulation, ii) some unascertained post-transcriptional modification(s) or mutation(s) in the mRNA and/or iii) post-translational modification(s) in the protein leading to their respective stabilization.

M541L substitution observed in the transmembrane domain in a solitary meningioma case (M16) has also been reported amongst healthy individuals [42,43]. This substitution was shown to be non-activating *in vitro,* though this observation may not fully reconcile with an *in vivo* scenario [44].

Taken together, it would be of interest to pursue whether the enhanced KIT expression in subset of meningioma cases acts as a catalyst in, complicit to or as a consequence of the meningeal neoplastic process. Further, it would be vital to determine whether KIT immunopositive meningioma cases, indeed have an activated oncoprotein. Owing to insufficient quantum of resected tissues, this aspect could not be pursued during the present study. Also, due to varying incidence rates of meningioma grades/sub-types, patient accrual and sample collection are slow processes. These limitations pose impediment for a study on fair distribution of all the pathologic subtypes.

## Conclusions

Conclusively, contrary to earlier reports, present study demonstrates KIT immunoexpression in a subset of human meningiomas. The data suggest that over-expression of the receptor in meningioma occurs without gene amplification and is associated with transcriptional activation. Continued work in this area would help elucidate the relative contribution of deranged KIT expression to the oncogenic pathways operative in this neoplasia, thus facilitating optimization of treatment modalities.

## Competing interests

The authors declare that they have no competing interests.

## Authors’ contributions

MS with guidance from SA and ANJ conceived and designed the study and performed the experiments. With the help of SA, MS analyzed and interpreted the molecular data. ANJ provided the patient tissues for the study. AA performed diagnosis and grading of resected tumor tissues; helped with analysis, interpretation and reporting of the immunohistochemical data and revising the manuscript. SA participated in the conception and design of the study, helped in drafting and revising the manuscript. All the authors read and approved the final manuscript.

## Pre-publication history

The pre-publication history for this paper can be accessed here:

http://www.biomedcentral.com/1471-2407/12/212/prepub

## Supplementary Material

Additional file 1 **Figure S1.** Detection of *KIT* transcripts by RT-PCR. (A) RT-PCR results of representative meningioma tumor tissues using primers specific to cytoplasmic domain of the *KIT*. (B) Confirmation of the quality of cDNA synthesis through RT-PCR using *ACTB* primers (also served as well loading control). Note the absence of amplicons in the no template control (NTC). NN denotes non-neoplastic. (TIFF 183 kb) Click here for file

Additional file 2 **Table S2.** Details of the primers used. Click here for file

Additional file 3 **Figure S2.** Agarose gel pictures verifying identity of the BAC clone RP11-586A2. PCR amplification for establishing the identity of BAC clone using primers specific to: (A) cytoplasmic; (B) transmembrane and (C) extracellular domains of the *KIT*. Note the absence of amplification in the no template control (NTC). The characterized clones were used to determine alterations of *KIT* in the neoplastic tissue by FISH. C denotes colony and NN, non-neoplastic. Click here for file

Additional file 4 **Table S2.** Copy number status of *KIT*, its signal intensity based on FISH and mutation analysis. Click here for file
