# Supplementary figures and images for: Expression of proto-oncogene KIT is up-regulated in subset of human meningiomas
Source: BMC Cancer. 2012 Jun 6;12:212. doi: 10.1186/1471-2407-12-212 (PMC3443037; doi:10.1186/1471-2407-12-212)

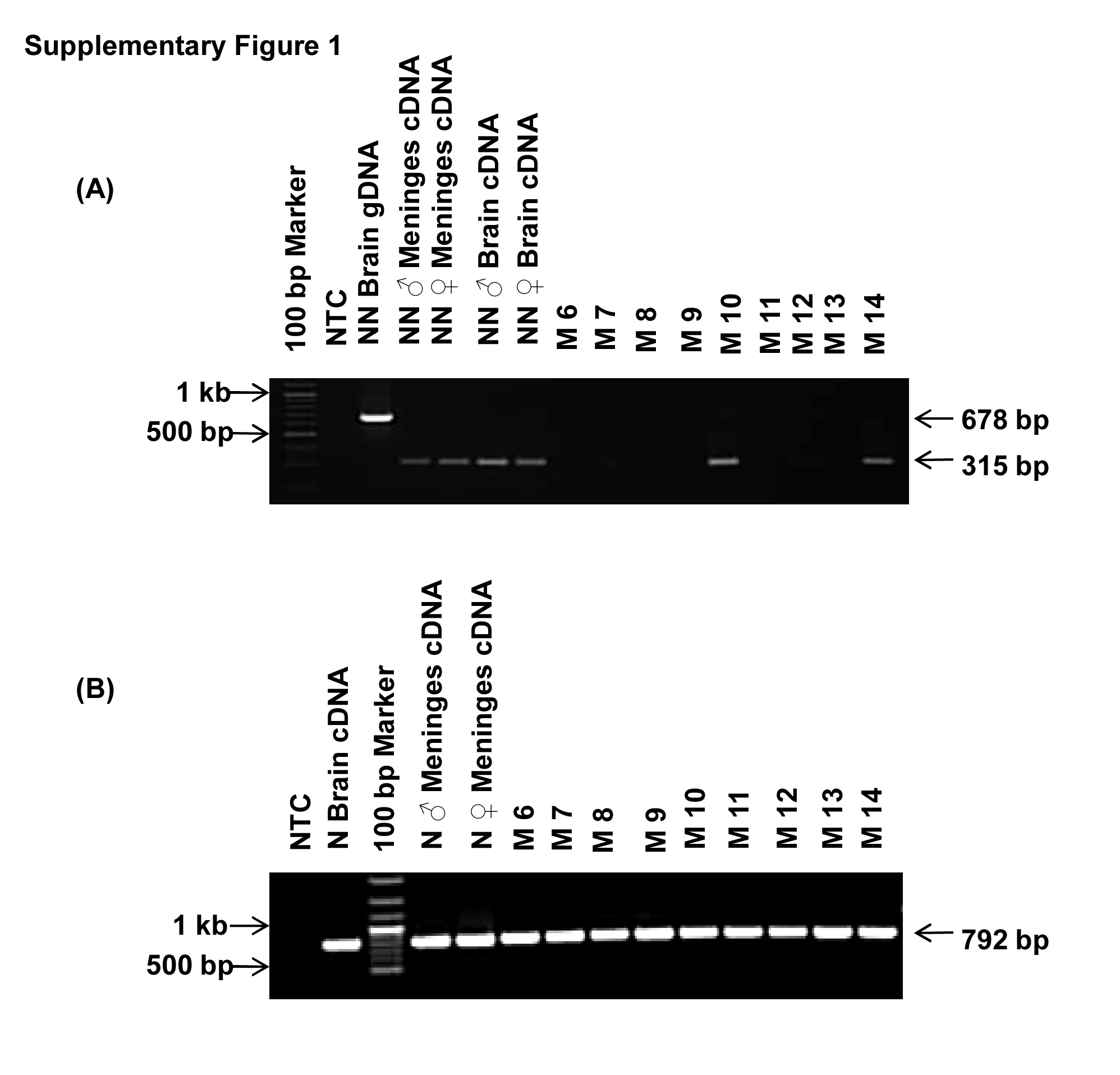

Supplement: Additional file 1 — Figure S1. Detection of KIT transcripts by RT-PCR. (A) RT-PCR results of representative meningioma tumor tissues using primers specific to cytoplasmic domain of the KIT. (B) Confirmation of the quality of cDNA synthesis through RT-PCR using ACTB primers (also served as well loading control). Note the absence of amplicons in the no template control (NTC). NN denotes non-neoplastic. (TIFF 183 kb) [file 1471-2407-12-212-S1.tiff]

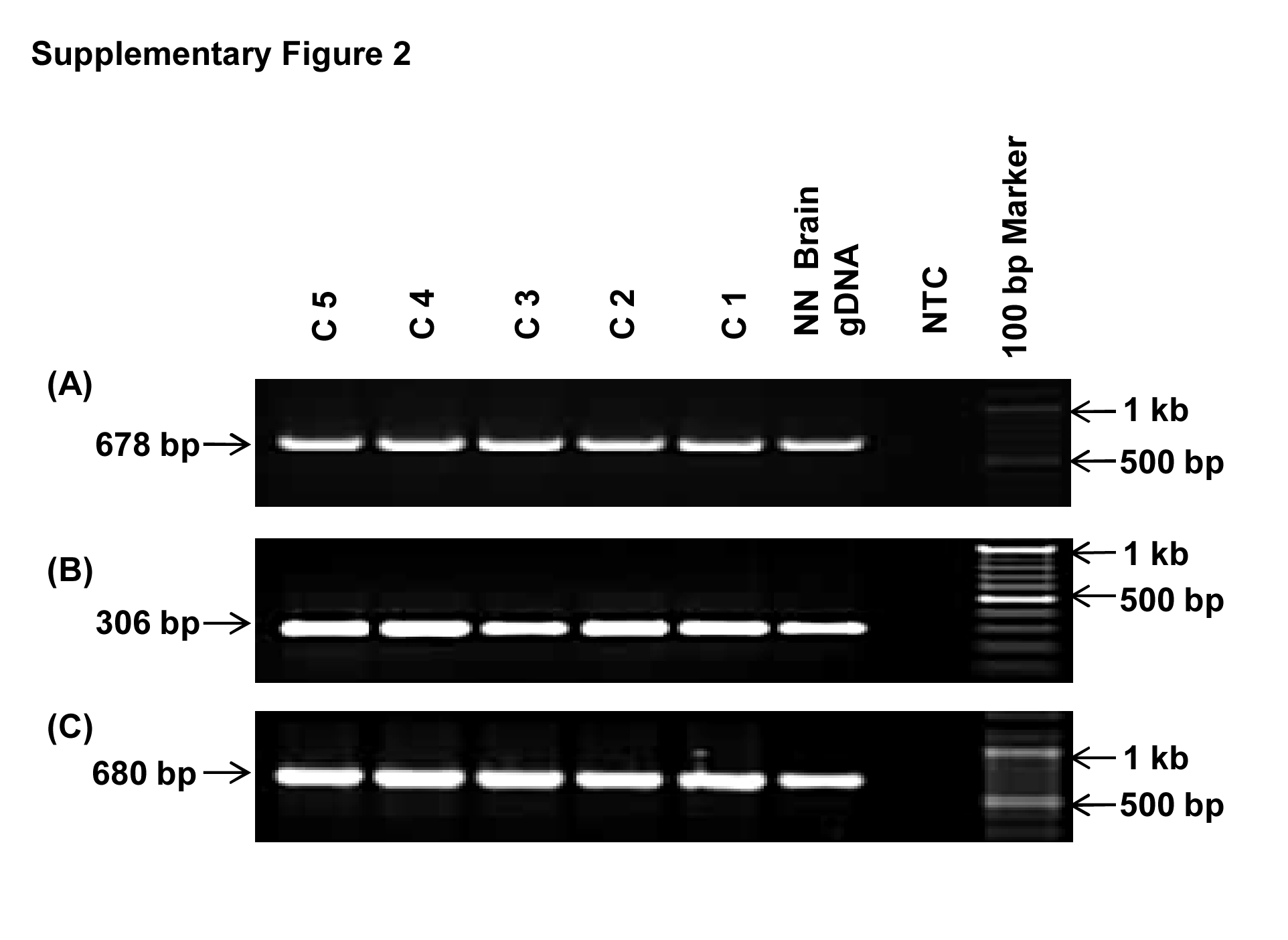

Supplement: Additional file 3 — Figure S2. Agarose gel pictures verifying identity of the BAC clone RP11-586A2. PCR amplification for establishing the identity of BAC clone using primers specific to: (A) cytoplasmic; (B) transmembrane and (C) extracellular domains of the KIT. Note the absence of amplification in the no template control (NTC). The characterized clones were used to determine alterations of KIT in the neoplastic tissue by FISH. C denotes colony and NN, non-neoplastic. [file 1471-2407-12-212-S3.tiff]
