# Supplementary material for: Expression of proto-oncogene KIT is up-regulated in subset of human meningiomas
Source: BMC Cancer. 2012 Jun 6;12:212. doi: 10.1186/1471-2407-12-212 (PMC3443037; doi:10.1186/1471-2407-12-212)
Supplement: Additional file 2 — Table S2. Details of the primers used. [file 1471-2407-12-212-S2.doc]

**Supplementary Table 1. Details of the primers used.**

|  | **Primer sequences (5’** → **3’)**  **Forward Reverse** | | **Amplicon**  **size (bp)** | **Annealing**  **temperature (^O^C)** | **MgCl_2_**  **concentration**  **(mM)** |
| --- | --- | --- | --- | --- | --- |
| 1. **Expression analysis (RT-PCR and RT-qPCR based)** | | | | | |
| ***ACTB*** | AGATGACCCAGATCATGTTTGAGA | CTAAGTCATAGTCCGCCTAGAAGCA | 792 | 61 | 1.5 |
| ***KIT* ^a^** | TAAAGGTAACAACAAAGAGCAAATCC | AGGTCAGAATCATCACAATAATGCA | 110 | RC | N.A. |
| ***KITLG* ^a^** | GGCAAATCTTCCAAAAGACTACATG | CTACCATCTCGCTTATCCAACAATG | 76 | RC | N.A. |
| ***GAPDH* ^a^** | GCCACATCGCTCAGACACCAT | ACCAGGCGCCCAATACG | 72 | RC | N.A. |
| 1. **Copy number analysis**ǂ | | | | | |
| ***KIT*** | Commercial pre-designed TaqMan assay (Hs02812715_cn; Applied Biosystems; designed on exon 5) | | 92 | RC | N.A. |
| ***RNase P*** | TaqMan Human RNase P detection kit (Applied Biosystems, P/N: 4316831) | | N.D. | RC | N.A. |
| 1. **Mutation analysis of *KIT* exons ^b^** | | | | | |
| **Exon 1** | AGTCAAGAGCGGGGAGAGAG | ACAGGCTTCGCCGAGTAGT | 415 | 57.8 | 1.5 |
| **Exon 9** | ATGCCACATCCCAAGTGTTT | TGAGTTTGATGACAGTATGGTGTG | 407 | 56.7 | 1.5 |
| **Exons 10 & 11** | GTTTGGGACTGAGTGGCTGT | CCCAAAAAGGTGACATGGAA | 440 | 56.7 | 1.5 |
| **Exons 12 & 13** | CTGCACAAATGGTCCTTCAA | AATCTAGCATTGCCAAAATCA | 453 | 55.9 | 1.5 |
| **Exon 17** | TGATTTTTATTTTTGGTGTACTGAA | TGTCAAGCAGAGAATGGGTACT | 299 | 55.9 | 1.5 |
| **D. Primers for different *KIT* domains used for BAC validation** | | | | | |
| **Extracellular** | TGTTTTTCTTGGCAGGCTCT | CGGTCTGGTTAGGCTTTGAG | 680 | 64.3 | 1.5 |
| **Transmembrane** | CACCCTGTTCACTCCTTTG | AACTCAGCCTGTTTCTGG | 306 | 59 | 1.5 |
| **Cytoplasmic** | TGGAAGGTTGTTGAGGAGATAAA | GGTGCAGGCTCCAAGTAGATT | 678, 315 **^c^** | 59 | 1.5 |

**^a^** Forward and reverse primers either span different exons, with large introns in between, or the 5' and 3ʹ portions of the primer lie on different exons, thus eliminating any chance amplification from the possible contaminating genomic DNA.

**^b^** Forward and reverse primers were designed from the intronic regions flanking the respective exons.

**^c^** Amplicon Size with gDNA is 678 bp and with cDNA is 315 bp).

RC, Recommended conditions; N.A., Not applicable; N.D. Not disclosed by vendor.
