# Supplementary material for: Expression of proto-oncogene KIT is up-regulated in subset of human meningiomas
Source: BMC Cancer. 2012 Jun 6;12:212. doi: 10.1186/1471-2407-12-212 (PMC3443037; doi:10.1186/1471-2407-12-212)
Supplement: Additional file 4 — Table S2. Copy number status of KIT, its signal intensity based on FISH and mutation analysis. [file 1471-2407-12-212-S4.doc]

**Supplementary Table 2. Copy number status of *KIT*, its signal intensity based on FISH and mutation details.**

| **Tissue**  **type** | **Case**  **id.^a^** | **Clinical diagnosis** | **KIT immuno-phenotyping ^a^** | ***KIT* copy no. assessment ^b^** | | | | ***KIT* mutation ^c^ (sequencing)** | |
| --- | --- | --- | --- | --- | --- | --- | --- | --- | --- |
|  |  |  |  | **qPCR** | | **Dual FISH (Tissue)** | |  |  |
|  |  |  |  | **Blood** | **Tissue** | ***KIT*/CEP4** | ***KIT*/Nucleus** | **Blood** | **Tissue** |
|  | **M10** | I, M | W | 2.29 | 2.31 | 1.327 | 1.945 | WT | WT |
|  | **M14** | I, T | Md | 1.96 | 2.25 | 1.242 | 1.873 | WT | WT |
|  | **M15** | I, M | W-Md | 1.85 | 1.59 | 0.892 | 2.071 | WT | WT |
|  | **M16** | I, T | W | 1.88 | 1.95 | **NE** | | **Ex 10, M541L, NSV** | |
| **Meningiomas** | **M21** | I, F | S | 2.00 | 1.72 | **NE** | | WT | WT |
|  | **M29 ^d^** | II, At | S | 2.29 | **1.17** | **0.408** | **0.891** | WT | WT |
|  | **M37** | I, T | W | 1.92 | 2.04 | 0.997 | 2.023 | WT | WT |
|  | M1 | I, T | N | 2.19 | 2.47 | – | – | – | – |
|  | M4 | I, M | NE | 1.75 | 1.64 | – | – | – | – |
|  | M19 | I, Ag | N | 2.08 | 2.09 | – | – | – | – |
|  | M33 | I, M | N | 2.09 | 1.89 | – | – | – | – |
| **NN Cerebellar tissue** | N.A. | N.A. | Md | **2.24** | **2.44** | **0.982** | **2.136** | **WT** | **WT** |

**^a^** KIT staining intensity: Weak (+); Moderate (++); Strong (+++). N, KIT negative (IHC & *KIT* copy no. for representative KIT negative samples has been shown).

**^b^** *KIT* gene copy no. relative to *RNase P* gene/diploid genome, aberrant copy numbers in bold.

**^c^** Samples with Exon- specific sequence variations in both, tumor and matched blood, are in bold. WT, Wild type; NSV, Non-synonymous variant; SV, Synonymous variant. The variants were observed in a

heterozygous pattern in all cases.

**^d^** Case M29 had 95% cells with loss of one *KIT* allele.

N.A., Not applicable; NE, Not evaluable; NN, Non-neoplastic; (–), Experiment not performed.
